# Promoting pedestrian ecomobility in Riyadh City for sustainable urban development

**DOI:** 10.1038/s41598-022-18183-y

**Published:** 2022-08-31

**Authors:** Ihab Katar

**Affiliations:** Department of Engineering Management (Construction Engineering Management Program), College of Engineering, Prince Sultan University, Riyadh, Kingdom of Saudi Arabia

**Keywords:** Civil engineering, Behavioural ecology

## Abstract

As the world is giving climate change a higher priority, several Coalitions are working on meeting a clean and green environment (SDG 13), focusing on new streets’ design in total urban development. A previous study discussed the pedestrian mobility status in Riyadh city, with attention to sustainable mobility, considering the pedestrians’ traveling patterns that use their cars rather than public transportation. This paper investigates the Ecomobility that seeks to promote the integration of passenging, cycling, wheeling, and walking. This approach will provide better health (SDG 3), clean air, mobility and accessibility, noise avoidance, greenhouse gas emission reduction, individual cost savings, and energy efficiency (SDG 11). It analyzes the pedestrians’ traveling modes in Riyadh city through a case study of the Prince Sultan University’s (PSU) community, then evaluates the pedestrians’ environment quality in the campus surroundings. Analysis used a web-based survey referred to the PSU people, including Students, Faculty, and Staff. On the other hand, the pedestrian’s environment quality was evaluated on-site built on well-constructed criteria. The assessment’s results addressed the factors influencing pedestrian choices and underlined the barriers to transformation to Ecomobility. They revealed that around 73% of the respondents expressed willingness to transform to ecomobility modes. About 52% of the survey sample preferred the public bus option. For the non-motorized options, students preferred cycling and wheeling modes, while faculty members and staff preferred walking. Based on conclusions, recommendations are proposed to foster pedestrian ecomobility for sustainable urban development in Riyadh city.

## Introduction

The Ecomobility is a cross-sectoral, global partnership, an environmentally friendly and socially inclusive way of transportation, seeking the promotion integration of cycling, walking, and passenging (Being a passenger in another's car or a carpool)^1^, and wheeling (wheelchairs, non-motorized scooters, walking aids, skates, push scooters, trailer, hand carts, shopping carts/ trolleys, carrying aids)^2^. In this section, the study introduces the main pillars that will build on them to reach the objectives. Passenging, energy efficiency, partnership for sustainable urban development, and streets’ design (with more focus as one of the core prerequisites of the methods) are discussed here. Although this research approaches the clean and green environment from the window of excluding the fossil-fuel motorized means of transportation, if there is still a necessity, passenging owns a pack of advantages over private cars. As a main passenging option, Carpooling has a lot of benefits to the environment, human health, and social lives, e.g., saving money, better for the environment, Convenience, etc., which are briefed below.

### Passenging

Being a passenger in another’s car or carpooling that increases efficiency. The concept becomes more apparent, listing the benefits of carpooling as follows^3^:

1. Saving money: sharing the cost of gas and parking, cutting expenses by nearly 50%; the more occupants in carpooling, the more saving, and is socially economical. Also, there will be a reduction in constructing new roads, maintenance, and air pollution-related health costs.
2. Better environment: fewer cars on roads mean reduced Greenhouse Gas (GHG) emissions and better air quality.
3. Better health: helps reduce some health risks for a human being, such as cardiovascular and respiratory diseases, allergies, and neurological effects.
4. Convenience: provides commuting Convenience, less stress, and added advantage of companionship while commuting.
5. Better commuting options: works better for people living where transit service may be non-existent or limited.

### Energy efficiency

A study was performed in China to reduce GHG; the study claimed that China targeted the peak around 2030^4^. However, it is expected that the peak may come earlier than expected, which is reflected in the CO_2_ emission (from coal consumption and fossil fuels) to have its peak also before 2030. Another study evaluated the economic impacts of GHG emission reduction on the Brazilian economy reached that different sectoral targets may balance environmental benefits with the possible financial losses incurred by taxation policy or emission permits^5^.

The energy efficiency studies touched key triggers to reduce carbon emissions, In parallel. An analytical study in China (2013) indicated that if greater intensity emission reduction measures were taken, the carbon emissions would reduce by 31.01 million tons by 2015 and 48.81 million tons by 2020^6^. The previous results indicated the average reduction flow per time.

### Partnership for sustainable urban development

Saving the environment is cooperative work that requires sharing experiences with other partners worldwide. The cooperation between the Gulf region and China in the last half of the decade is an example of a global partnership. The Gulf region has achieved a success story, economically and socially. Following a smart investment arrangement of natural resources, the region has succeeded in attracting and retaining international experience, therefore overcoming the discrepancy between its vast economy and small population-base lacking the instantly needed technical skills. The region has collected considerable wealth during the process, financially and else^7^.

The primary purpose of Ecomobility, mentioned previously, is enhancing the opportunity for sustainable urban development. Also, it complies with John Elkington’s triple bottom line (TBL), or what is often referred to as the three “P’s”: people, planet, and prosperity^8^. Likewise, worldwide, large cities are applying guidelines to guarantee that environment, economics, and sociality are at the lead of urban design. The elevation of healthier streets has formed new chances for social and commercial interaction and more comprehensive outcomes^9^. Overall, it is the transformation of the earlier global Coalitions of Ecomobility that aims at engaging public and private sectors, promoting and advocating Ecomobility at a worldwide level in Industrialized and developing countries.

### Streets’ design

A potential approach to fostering pedestrian Ecomobility is the streets’ design, which is a core focus of this research. Urban spaces are a vital city part, forming the basic structure of public life. Specific urban planning and design criteria make these spaces “quality public spaces”^10^. In this perspective, determining and evaluating these criteria will transform those public urban spaces into quality spaces. The last research adopted criteria that came to a head in the relevant reviews and were accepted by most researchers. Also, in the current research, the study chose following these criteria since they have been collected and validated by several researchers over decades (around 17 references; Table 1), and cover the possible aspects of successful streets; they will be presented and used in the next section (Methods).

**Table 1:** Respondents’ statistics (N = 257).

Main characteristics		Students (N = 174)	Faculty members (N = 55)	Staff (N = 28)
Gender	Female	91 (52.3%)	27 (49%)	9 (32.1%)
	Male	83 (47.4%)	28 (51%)	19 (67.9%)
Physical disability	No	168 (96.6%)	52 (94.5%)	27 (96.4%)
	Maybe	5 (2.9%)	1 (1.8%)	0 (0.0%)
	Yes	1 (0.5%)	2 (3.7%)	1 (3.6%)
Age	Less than 24	166 (95.4%)	10 (18.2%)	19 (67.9%)
	From 24 to 50	8 (4.6%)	33 (60%)	7 (25%)
	Over 50	0 (0.0%)	12 (21.8%)	2 (7.1%)
Nationality	Saudi	132 (75.9%)	6 (10.9%)	19 (67.9%)
	Non-Saudi	42 (24.1%)	49 (89.1%)	9 (32.1%)
Distance to the campus	Less than 1 km	5 (2.9%)	0 (0.0%)	0 (0.0%)
	From 1 to 3 km	18 (10.3%)	7 (12.8%)	1 (3.6%)
	From 5 to 8 km	55 (31.6%)	24 (43.6%)	11 (39.3%)
	Longer than 10 km	96 (55.2%)	24 (43.6%)	16 (57.1%)

A noteworthy point is the gap between municipal policies and the guidance provided to street design decision-makers. A study across cities in the United States highlighted some issues regarding the mechanism of developing policies related, as they aim to challenge auto-centrical street design standards in favor of “complete streets,” which are safe for users of all abilities. Also, they address the demands of non-motorized street users and sustainable transportation. Moreover, those policies do not lead to the negotiation of tradeoffs among users inside the street right-of-way; They are broad and defer to optimistic safety goals accommodating all user types equally without recognizing the accommodation’s implicit hierarchy^11^.

Also, the transformative potential of experimentations proposed attaining “streets for people” rather than “streets for traffic” remains under-investigated. There is little to no comparative assessment of already present trials and no critical reflection on their explicit added value for systemic change. While street research aims to create basically diverse arrangements of urban mobility, their potential as triggers of a larger systemic change is blurred^12^.

In the age of autonomous vehicles (AVs), a study reached that with the promise of Avs, which will use less street-right of way and uncouple parking from land uses in the cities; it is time to take back streets and make them serve people first, arranging cycling, walking, and transit. This action will take place precisely in the design process for livable streets. Also, it is time to act and make sure that designers and planners can get at the forefront of the rapidly changing technologies of vehicles^13^.

The street design process had a different approach through medical and psychological professionals. According to the Irvine-Minnesota audit, expert observers calculated street users for four streets, which differed in walkability. From 7 am to 7 pm all days, the whole streets had significant quadratic trends of increasing followed by decreasing use. Furthermore, the two most walkable streets showed substantial linear increases in users across the day. Part of a street’s identity is its temporal activity rhythm, and both walkability and rhythms can report to urban design and restoration^14^. The study, through its test, searched what makes streets and neighborhoods walkable and found that more walkable streets had more users overall and linear increases in use starting from morning to early evening.

This paper focuses on Ecomobility in Riyadh city streets that promote the integration of passenging, cycling, wheeling, and walking. A previous study discussed the pedestrian mobility status in the same city, focusing on the transformation to sustainable mobility. That research case study was applied to the PSU community and argued that the current mobility is unsustainable, as it profoundly relies on privately-owned cars run with fossil fuel. It unveiled that a substantial percentage of the survey sample (72%) traveled by car from home to the campus. However, the assessment results showed that the transformation to sustainable mobility is expected soon by launching the new mega projects related to public transportation like Riyadh Metro and busses, which is considered a key indicator of sustainable mobility^15^.

**Figure 1:**
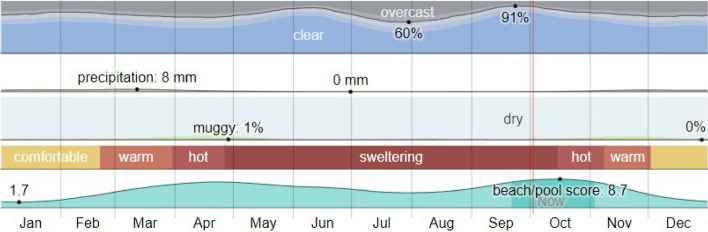
The climate in Riyadh^16^.

**Figure 2:**
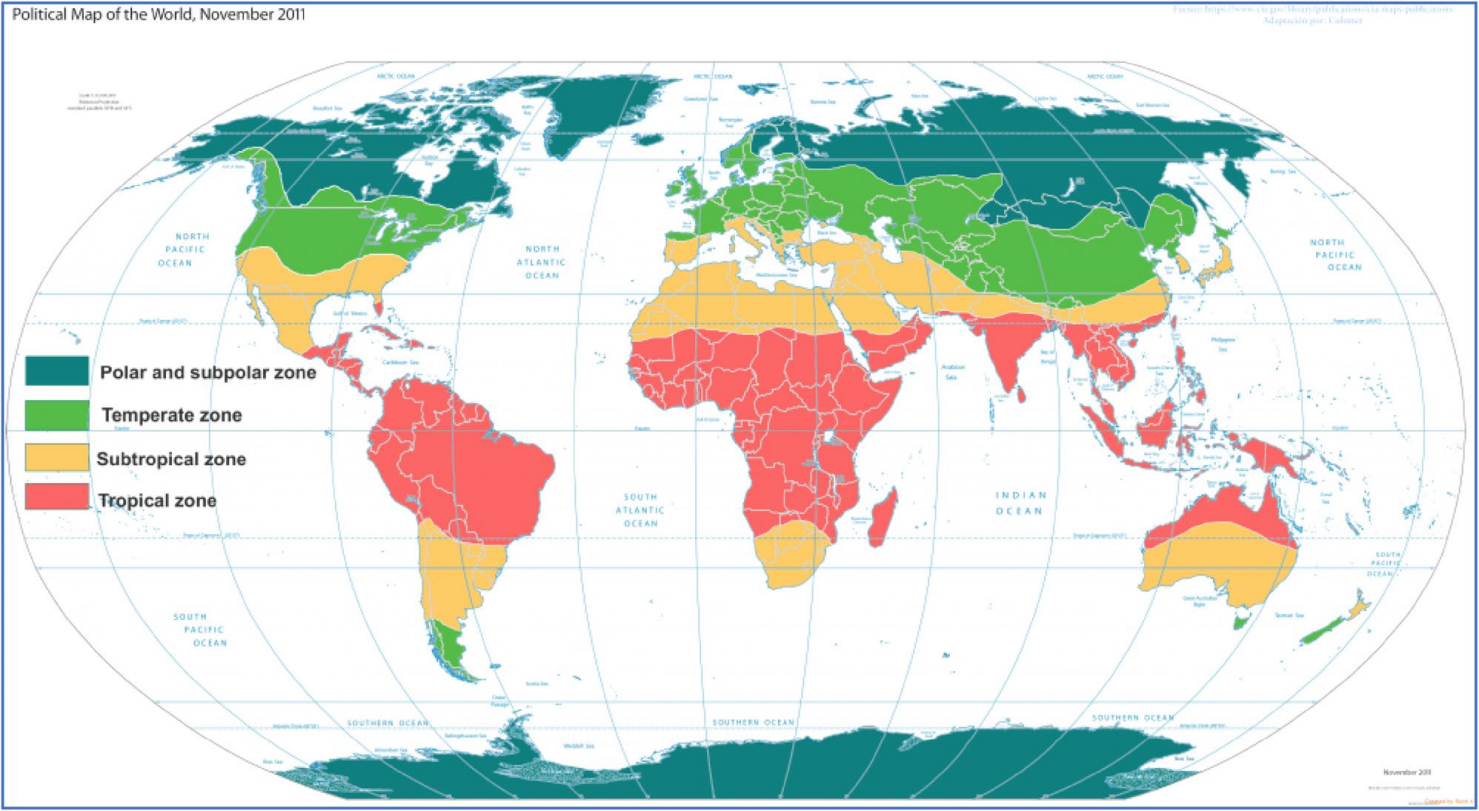
World climate zones^17^.

Riyadh city in Saudi Arabia is a part of a hot-weather zone (almost 7 months a year: April–October), as seen in Fig. 1^16^. As a result, studying the pedestrian thermal comfort affected by street design is a core point. Almost similar conditions were present in a case study in the Australian North Melbourne (southeast) at street level for pedestrians (Subtropical Zone), as seen in Fig. 2^17^. That study assessed modeled existing and future scenarios for different street profiles and the consequences of microclimatic parameters and thermal comfort. The target was to assist urban planners in developing policies that can efficiently reduce the exposure to heat stress at the pedestrian level^18^.

A comparison study was also applied to two gulf cities that share the hot weather: Dubai and Abu Dhabi. It compared the efficiency of the early suburbs and the newer ones to provide quick and direct access to destinations (connectivity). The study argued that better connectivity is required for usefulness in hot, dry regions. In this regard, it explored how the abandoned system of alleys could be cultivated to enhance connectivity efficiency^19^.

In residential streets, developers' width is not a choice but somewhat a constraint enforced through planners' concluded subdivision standards. Residents can generally compromise by choosing smaller yards or homes in a swap for other facilities or a lower price, but they cannot select a smaller street^20^.

Recently, in Riyadh city, a study highlighted that the rising fuel prices would create a positive atmosphere for implementing the complete streets’ concept, defined as streets’ design that can safely accommodate all transport modes for all society segments. It includes, but is not limited to, public transportation, humanizing neighborhoods, and promoting walking as a healthy lifestyle^21^.

### Research problem

Lacking the dependency on the Ecomobility traveling modes (as mentioned in the results) leads to poor health conditions, air pollution, noise, high greenhouse gas (and CO_2_) emissions, higher expenses, and excessive energy consumption.

### Research questions

The study is seeking through its parts to answer the following questions:

1. What are the reasons behind the lack of using the ecomobility means of transportation by the community?
2. What are the community responses for the transformation to ecomobility if the reported barriers are removed or minimized?

## Methods

The methods in this section were developed following applicable global standards’ guidelines and rules.

This study investigates the ecomobility in Riyadh city through a case study of Prince Sultan University (PSU) and its surrounding district environment. The assessment achieved is divided into two sections. First, the traveling modes of the PSU community are collected and analyzed using a web-based survey. Second, a field assessment is conducted to evaluate the environment’s pedestrian quality around the university campus.

### Traveling modes and individuals’ behavior

A web-based survey has been designed and employed as a tool to gather data regarding individuals’ behavior and their transportation mode choice. This survey was directed to the PSU community: students, faculty, and staff, among male and female campuses. The survey was applied between October and November 2021 (where the physical attendance on the PSU campus was resumed with strict health precautions within the Covid-19 Pandemic extended period: wave 4). Consequently, the web-based survey procedure has been employed due to health constraints as the PSU was still applying hybrid classes and meetings. This practice was effectively adopted in several previous studies^22–24^. The Institutional Review Board (IRB), according to PSU rules for similar surveys, reviewed the content and structure to guarantee that all privacy terms and conditions are considered. Therefore, the survey form was delivered to the PSU academic system and was approved. The survey was structured of 18 questions, divided into four different parts. The first part contains general demographic questions, e.g., age, gender, PSU position (student, faculty, or staff), nationality, traveling distance to the PSU campus, and any reported physical disability. The second part contained questions about the primary transportation modes used for arrival and departure. The provided choices included: private cars (Fuel/Electric or hybrid), bus (public/private), car-sharing, cycling, wheeling, and walking. In the third part, participants were asked to identify the most probable reasons for using their choice of transportation modes, where checking boxes were allowed if they had several reasons. By the end of the third part, participants were asked about their future readiness to alter their traveling modes to minimize the barriers. In the fourth part, respondents were asked to identify different barriers preventing them from using other environmentally friendly transportation alternatives, e.g., passenging cycling, wheeling, and walking, to commuting to the campus.

**Figure 3:**
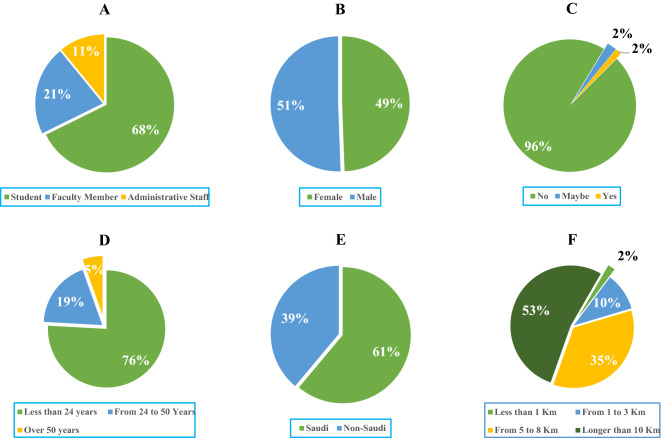
Main characteristics of the respondents (N = 257). (**A**) Role in the University. (**B**) Gender. (**C**) Physical disability. (**D**) Age. (**E**) Nationality. (**F**) Distance to the campus.

After data collection, the survey responses were analyzed using the Statistical Package for the Social Sciences (SPSS). Survey forms were distributed to the whole PSU community, and 257 responses were collected through the survey. The survey response rates obtained were: 174 from students (6048), 53 from Faculty members, one programmer and one researcher (435), 24 from staff, and four from higher management (391). This sample represents 2.9%, 12.6%, and 7.2% of the PSU students, faculty, and staff, respectively, which means around 7.6% of the PSU community. The main characteristics of the respondents are shown in Fig. 3. The sample was divided into three groups: 68%, 21%, and 11% of the sample of students, faculty members, and staff, respectively. Table 1 explains the characteristics of each group. Most students were below 24 years of age, and most adults above 24 were faculty members and staff.

### Pedestrian’s environment quality

The study examined the pedestrian mobility modes to assess the environment quality. Also, it addressed the pedestrian’s concerns that affected their behavior inside the study area. The environment’s field assessment involved exploration, pilot survey, and design evaluation based on the design standards and codes^25,26^. According to Riyadh Royal Commission’s standards for development, Riyadh city streets are classified into four types based on their functionality (Types P, M, R, and A). The first type is the mixed residential roadways (Type “M”), and the second is the Pedestrian/Public Transport Oriented streets (Type “P”). The third and fourth types are the Residential “R” and Access “A” road types. This field study only included the types “P” and “M,” as seen in Fig. 3, as a sample because they own the priorities of enhancement in governmental planning. On the other hand, “R” and “A” street types were excluded and added to the study limitations for future research.

**Figure 4:**
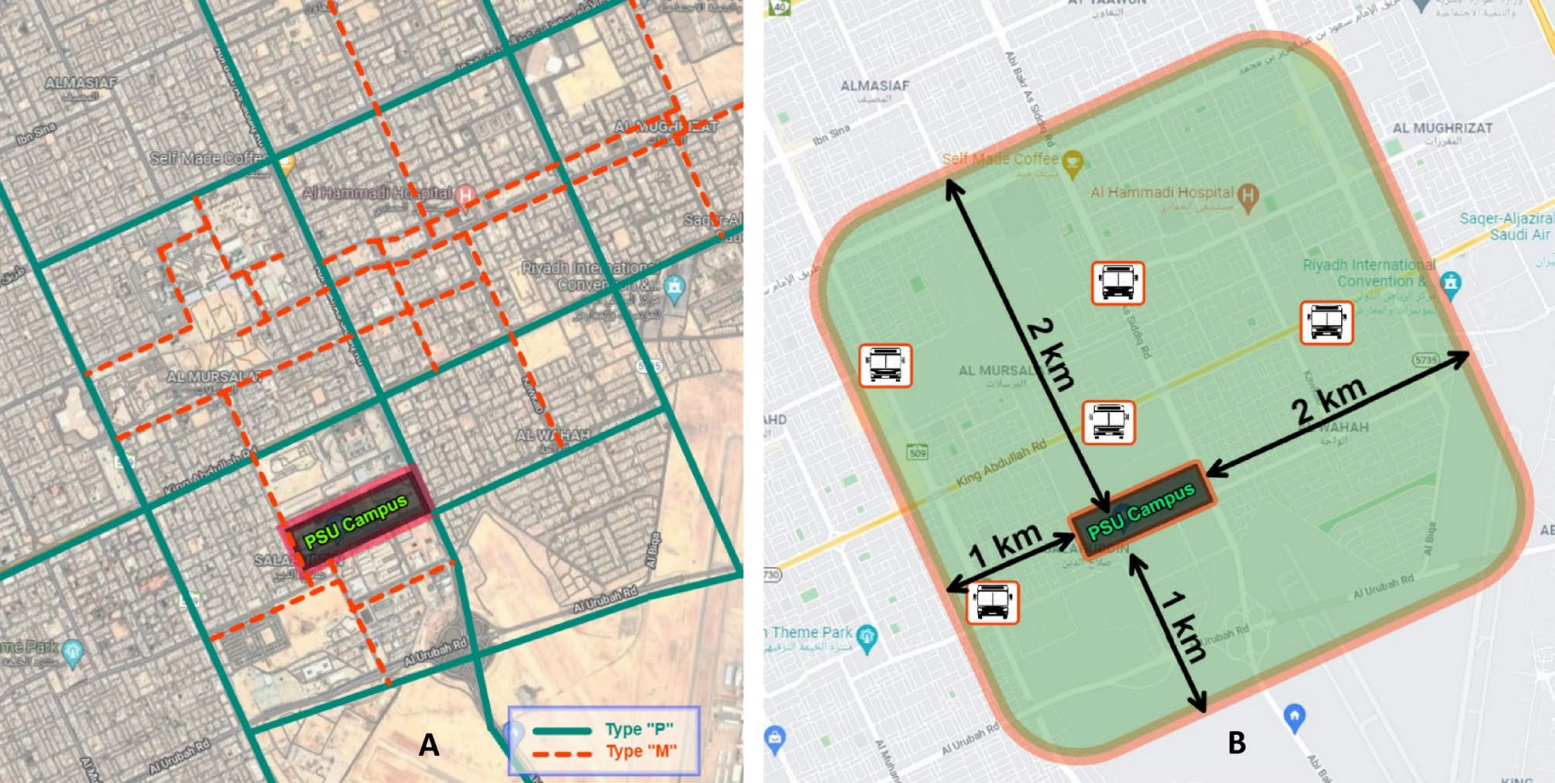
Study zone embraced in the field assessment. (**A**) Selected roads along routes that pedestrians could use for traveling to PSU. (**B**) Study zone dimensions and partially operated bus stations (PIXLR X, https://pixlr.com/x/).

**Figure 5:**
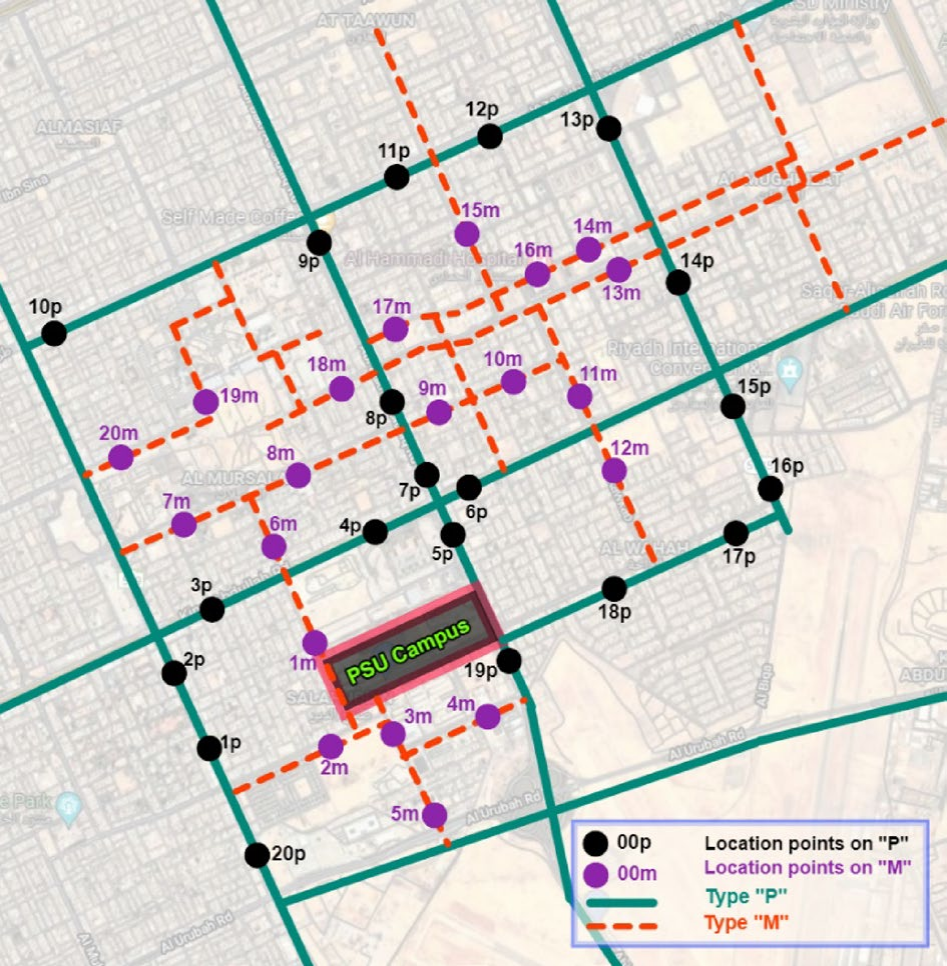
Selected location points of the field assessment, distributed among both types “P” and “M” (PIXLR X, https://pixlr.com/x/).

About 9 km^2^ (3 × 3 km) around the PSU campus was the study zone embraced in this assessment, as seen in Fig. 4. Also, the locations of bus stations (partially operated) are specified within the study zone in the previous Figure. 40 location points were chosen for the selected streets’ assessment; 20 points were located on the Type “P” streets and 20 points on the Type “M,” as seen in Fig. 5. the location point selection considered the diversity and balanced distribution.

Many assessment methods and criteria have been developed worldwide, but few have tested the obtained results’ authenticity. As introduced in the previous section, regarding the chosen criteria for street assessment, Table 2 shows the main and sub-criteria used in this study, including (5) items for the main criteria and (13) items for the sub-criteria, associated with a brief description of each.

**Table 2:** Description of main and sub-criteria of street assessment [Umut Dogan 2021].

Main criteria	Sub-criteria	Description
**1. Continuity:** The continuity of the streets as a physical condition^29–32^	(a) Pedestrian cont	If a pedestrian can move between two points of a street without interruption, this corresponds to a high score
	(b) Access cont	Continuity of access of pedestrians and all motor/non-motor vehicles in reaching their desired destination: if the desired point can be reached comfortably and without interruption, a high score is obtained
	(c) Function cont	Services offered along the street support each other. Also, it is the existence of the functions provided to the users without being disconnected from each other
**2. Diversity:** The differences created by a space and its users. It also expresses the visual richness of a space^29–36^	(a) Vitality	Liveliness: As the diversity of a street increases, it also affects its vitality because diversity will bring along various functions and users. Also, the vitality of a street means that the space is of higher- quality^37,38^
	(b) Multilayeredness	Physical and functional surfaces offer alternative uses; Diverse interfaces, negative and positive functions, surprising & fun places and events, flexible altering activity environments, etc
	(c) Function Variety	The richness of the functions of the street
**3. Esthetic:** Physical dimensions that affect the perception and satisfaction of pedestrians^29,30,32,35,39–41^	(a) Visual Qualities	The success of the composition in street scenes and the way it is conveyed to the user is an indicator of the visual quality
	(b) Attractiveness	The richness of street scenes brings in the Attractiveness of the space
	(c) Imageability	The quality of a physical environment that creates a strong image in the observer
**4. Comfort:** Related to the high or low standard of service offered by the urban environment^29–32,34,42–44^	(a) Safety	Roads are free of vehicle traffic, transport safety, removal of risky environmental elements, crime rate, etc
	(b) Access comf	The presence of pedestrians, bicycles, strollers, disabled people, the elderly, children, etc. and all other users in the street in a comfortable way
**5. Scale:** The size, location, distance, and depth of the objects based on some physiological and psychological effects^40,41,45–47^	(a) Human	Highly dependent on physical features such as city furniture, building details, and walking distance
	(b) Environmental Compliance	The relationship between all physical formations in space and environmental data; natural environments (such as climatic, topographic, geological, and vegetation, etc.) are factors that affect the urban quality

A pilot field assessment is utilized to apply the 5-criteria layout on the selected streets along routes that pedestrians could use for traveling to the PSU campus, as seen in Fig. 3. 13 sub-criteria are considered in the assessment. Based on this layout, an expert panel was assigned to assess each of the selected points. The evaluation implemented the 1–5 score weight scale (Likert type). A rank was given for each point based on satisfaction and readiness to meet design criteria. The relative importance index (RII) is used in this assessment to weigh the criteria according to their relative importance. RII is a practical, commonly used tool to focus on criteria assessed on Likert-type scales. The following formula determines RII for each of the thirteen sub-criteria items^27^.

1 $${\text{Relative Importance Index }}\left( {{\text{RII}}} \right) = \sum \frac{W}{A*N}$$

where W, is the weight given to each item by respondents (The experts), ranging from 1 to 5, with one implying the least and five the highest as follows:

(5): Excellent for mobility; (4): Good for mobility; (3): Passable for mobility but requires minor attention; (2): Insufficient for mobility and requires some attention; (1): Insufficient for mobility and requires substantial attention. A: is the highest weight (according to 5 points Likert- type scales). N: total number of respondents (The experts).

Five importance levels (IL) were aligned with the calculated RII values^28^ and the decisions due to the mobility status as given in Table 3. The mobility status that meets the calculated RII and the aligned IL are also addressed in the same Table. Therefore, RII was calculated for each of the thirteen sub-criteria.

**Table 3:** RII values-IL levels-mobility status and decisions.

RII Values	IL		Mobility status and decisions
0.8 ≤ RII ≤ 1	High	H	[5]: Excellent for mobility
0.6 ≤ RII ≤ 0.8	High-Medium	H-M	[4]: Good for mobility
0.4 ≤ RII ≤ 0.6	Medium	M	[3]: Passable for mobility but requires minor attention
0.2 ≤ RII ≤ 0.4	Medium–Low	M-L	[2]: Insufficient for mobility and requires some attention
0.0 ≤ RII ≤ 0.2	Low	L	[1]: Insufficient for mobility and requires substantial attention

### Institutional approval of the used experimental protocol

The experimental protocol followed in this research was approved by the Institutional Review Board (IRB) of Prince Sultan University (PSU), and the approval document is provided with the submission set.

### Methods’ guidelines and regulations

The methods used in this research adhered to the standards of scientific research relevant to the study's nature, including the performed survey to the study sample and the involvement of the expert panel (a copy of the survey is provided with the submission set).

### Consent of the participants

Informed consent was obtained from all participants (Please note that the minimum age within-study sample is above 16 since they are university students (after high school), faculty members, and staff. All participants from the study sample were acquired to check the box of the following statement: “I have read and accepted the terms of use and privacy policies,” which include the consent form (attached in the submission set).

## Results

The first assessment section of this study was primarily directed to discovering the PSU community’s traveling modes to the campus. Also, the second assessment section was mainly engaged with evaluating the pedestrian environment quality around the PSU campus per the 5-Criteria model. The assessment results will be discussed in the next section, counting respondents' chosen primary traveling modes, factors affecting the traveling modes, barriers to using environmentally friendly and socially inclusive ways of transportation, and the quality evaluation results of the pedestrian environment. Furthermore, the results highlight the respondents’ percentages who are willing to switch to ecomobility modes to meet the clean and green environment requirements, in case of securing the basic needs in the street design in Riyadh city and particularly around PSU according to their stated barriers.

According to the survey and street assessment, the main findings show the pedestrians’ high dependency on private fossil-fuel cars, as mentioned in “Results /Principal transportation modes” and “Discussion /Principal transportation modes”. Also, there was a readiness to transform to the ecomobility modes by part of the pedestrians depending on the probability of removing or minimizing the barriers preventing them from using other environmentally-friendly alternatives. The last information was stated and discussed in “Results /Willingness to transform to ecomobility modes”, “Results /Assessment of street’s environment quality”, “Discussion /Willingness to transform to ecomobility modes”, and “Discussion /Assessment of environment’s quality of streets”, respectively.

### Principal transportation modes

**Figure 6:**
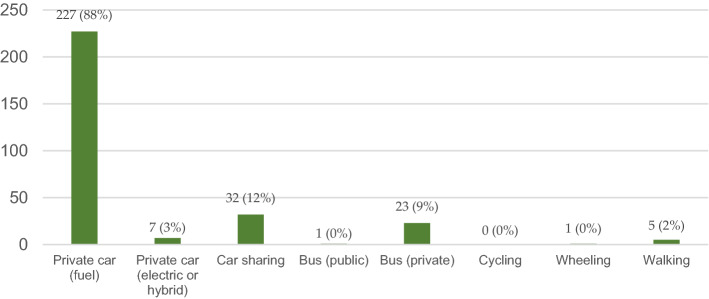
Principal mean/s of transportation to the campus.

The community’s principal transportation modes used to travel to the campus are given in Fig. 6. According to the web-survey results, 91% of the respondents drive to the campus (fuel and electric or hybrid cars). The fuel cars were the most used transportation mode by the students, faculty, and staff (96.6%, 60%, and 92.9%, respectively), as given in Table 4. Private bus (university bus), and car-sharing, were the second most used transportation modes (around 21%), as seen in Fig. 6; also, walking was chosen as a transport mode by 2% of the respondents. Table 4 indicates that walking to the campus is more popular with the students (11.5%) and faculty members (6.1%) than among staff, although the traveling distances were less than 1 km for one student, 1–3 km for one faculty member, and 5–8 km (relatively long walking distance) for one student and one faculty member. On the other hand, all respondents did not select cycling mode, and only one student chose wheeling. The principal transportation modes analysis by gender (Table 5) indicated that males and females are extensively dependent on the private car (fuel or electric/hybrid; 88.5% for males, and 92.8% for females). On the other hand, the public bus use was limited (only one male student; 0.8%), but the sample uses the private bus provided by the University since its availability (7.7% and 10.2% for male and female, respectively). The analysis presented for the choice of the principal transportation mode based on the traveling distance in Table 6 showed that only 20, 3.8, and 2.2% of the respondents (5, 26, and 90) selected the walking mode travelled to less than 1, 1–3, and 5–8 km, respectively. The private car was the primary choice for the respondents traveling longer than 10 and 5–8 km (94.1 and 83.3%), respectively. The high dependency on driving is justified for long distances, but it was not justified for short distances: less than 1, and 1–3 km (80 and 92.3%), respectively. This finding contradicts other results: the choice of transportation mode depends on the travel distance more than the age or position^48,49^.

**Table 4:** Principal transportation modes used to travel to the campus.

Mode\Group	Students	Faculty	Staff
N	174	55	28
Private car (fuel)	168 (96.6%)	33 (60%)	26 (92.9%)
Private car (electric or hybrid)	5 (28.7%)	2 (6.1%)	0 (0.0%)
Car sharing	21 (12%)	8 (24.2%)	1 (3.6%)
Bus (public)	1 (0.6%)	0 (0.0%)	0 (0.0%)
Bus (private)	1 (0.6%)	20 (60.6%)	2 (7.1%)
Cycling	0 (0.0%)	0 (0.0%)	0 (0.0%)
Wheeling	1 (0.6%)	0 (0.0%)	0 (0.0%)
Walking	2 (11.5%)	2 (6.1%)	0 (0.0%)

**Table 5:** Principal transportation modes by gender.

Mode\Group	Male	Female
N	130	127
Private car (fuel)	114 (87.7%)	111 (87.4%)
Private car (electric or hybrid)	1 (0.8%)	6 (5.4%)
Car sharing	17 (13.1%)	15 (11.8%)
Bus (public)	1 (0.8%)	0 (0.0%)
Bus (private)	10 (7.7%)	13 (10.2%)
Cycling	0 (0.0%)	0 (0.0%)
Wheeling	1 (0.8%)	0 (0.0%)
Walking	1 (0.8%)	3 (2.4%)

**Table 6:** Principal transportation modes by traveling distance.

Mode\Group	< 1 km	1–3 km	5–8 km	> 10 km
N	5	26	90	136
Private car (fuel)	3 (60%)	23 (88.5%)	74 (82.2%)	124 (91.2%)
Private car (electric or hybrid)	1 (20%)	1 (3.8%)	1 (1.1%)	4 (2.9%)
Car sharing	1 (20%)	4 (15.4%)	9 (10%)	16 (11.8%)
Bus (public)	0 (0.0%)	0 (0.0%)	0 (0.0%)	1 (0.7%)
Bus (private)	0 (0.0%)	1 (3.8%)	11 (12.2%)	11 (8.1%)
Cycling	0 (0.0%)	0 (0.0%)	0 (0.0%)	0 (0.0%)
Wheeling	0 (0.0%)	0 (0.0%)	1 (1.1%)	0 (0.0%)
Walking	1 (20%)	1 (3.8%)	2 (2.2%)	0 (0.0%)

### Factors controlled choices

The respondents’ reported reasons for their primary transportation mode choices are summarized in Tables 7, 8, 9, 10. As shown in Table 7, the most considered reason for driving to the campus was Convenience, as reported by 59.8%, 38.2%, and 32.1% of students, faculty, and staff, respectively. Also, long-distance between home and campus was the second reason for driving, as reported by 50.6, 21.8, and 21.4% (for the previous order). Besides, the need to perform other activities more than reaching the campus or home was the third reason for driving, as reported by 37.9, 21.8, and 17.9% (the previous order). Each respondent's group evaluated the car sharing and the private (University) bus differently (Tables 8 and 9). The staff was the minor group to use car-sharing for not owning a car or driving license (only 3.6%). The first higher dependency on car sharing was the students’ group, where 21.3% gave different reasons for using this choice. The second higher group was the faculty members, who provided various reasons for using this option (16.4%). Table 9 shows the dependencies on the private bus, where the faculty members’ group had the highest users (45.4%) over students and staff (0.6 and 10.7%), respectively.

**Table 7:** Reasons for choosing a private car (fuel, electric/hybrid).

Mode\Group	Students	Faculty	Staff
N	174	55	28
Convenience	104 (59.8%)	21 (38.2%)	9 (32.1%)
Perform other activities more than drives to and from the campus	66 (37.9%)	12 (21.8%)	5 (17.9%)
The long distance between my home and the campus	88 (50.6%)	12 (21.8%)	6 (21.4%)
Unavailable transportation alternatives to the campus	69 (39.7%)	16 (29.1%)	8 (28.6%)
Inconvenient transportation alternatives to the campus	56 (32.2%)	11 (20%)	5 (17.9%)

**Table 8:** Reasons for choosing car sharing.

Mode\Group	Students	Faculty	Staff
N	174	55	28
Temporary unavailability of a private car due to maintenance issues	13 (7.5%)	1 (1.8%)	0 (0.0%)
Inconvenient driving in traffic jams	4 (2.3%)	1 (1.8%)	0 (0.0%)
The long distance between my home and the campus	1 (0.6%)	3 (5.5%)	0 (0.0%)
Have no other serious activities to perform during the ride	3 (1.7%)	0 (0.0%)	0 (0.0%)
Not owning a car or don't have a driving license	12 (6.9%)	3 (5.5%)	1 (3.6%)
Inconvenient public transportation services	4 (2.3%)	1 (1.8%)	0 (0.0%)

**Table 9:** Reasons for choosing private bus.

Mode\Group	Students	Faculty	Staff
N	174	55	28
I'm not paid a transportation allowance as the university secure such rides	0 (0.0%)	1 (1.8%)	0 (0.0%)
The university arranges, frequently, other group rides for shopping	0 (0.0%)	4 (7.3%)	1 (3.6%)
Not owning a car or don't have a driving license	1 (0.6%)	7 (12.7%)	0 (0.0%)
Convenience	0 (0.0%)	13 (23.6%)	2 (7.1%)

**Table 10:** Reasons for choosing walking.

Mode\Group	Students	Faculty	Staff
N	174	55	28
The short distance between my home and the campus	2 (1.1%)	2 (3.6%)	0 (0.0%)
Practice a kind of sport activity at the same time of reaching the campus to save workout time	0 (0.0%)	0 (0.0%)	0 (0.0%)
All primary services and facilities are available	0 (0.0%)	0 (0.0%)	0 (0.0%)

### Barriers of the ecomobility modes

In the survey, participants were asked to identify the barriers to using alternative clean and green means of transportation. The barriers received from the respondents are summarized in Table 11. The weather conditions (hot, cold, rainy, sandstorm, etc.) were the main reason for not using these modes, as stated by 59.8, 49.1, and 60.7% of students, faculty members, and staff, respectively. Trip time was also reported as a major barrier to such modes, as reported by 44.3, 36.4, and 25% (for the previous order). Likewise, the long walking time to the campus was a significant reason to avoid this mode, as stated by 44.8, 32.7, and 25% (the previous order). Unavailable public bus stops near the home were also a substantial reason for not using this option, considering the partial initiation of the bus project, as reported by 42.5, 47.3, and 53. 6% (the previous order). On the other hand, safety and security reasons were significant barriers, as mentioned by 40.8, 32.7, and 35.7% (the previous order). For the walking and cycling modes together, 37.9, 29.1, and 14.3% (the previous order) mentioned avoiding those options due to the unavailability of bike lanes or continuous sidewalks. Almost the same reason’s weight went to car ownership, as stated by 40.2, 23.6, and 46.4% (the previous order). The following barriers came in the second level of importance (The need to offer rides to other family members or colleagues & the need to carry goods), as stated by (21.8, 7.3, and 17.9% & 14.9, 18.2, and 17.9%) (the previous order). The coming reported barrier is secondary: the lack of primary services and facilities or the physical disability arrangements reported by 8, 7.3, and 7.1% (the previous order).

**Table 11:** Barriers to using alternative clean and green means of transportation.

Mode\Group	Students	Faculty	Staff
N	174	55	28
Trip time	77 (44.3%)	20 (36.4%)	7 (25%)
Unavailable public bus stops near to my home	74 (42.5%)	26 (47.3%)	15 (53.6%)
Inconvenience	81 (46.6%)	20 (36.4%)	10 (35.7%)
No available bike lanes or continuous sidewalks	66 (37.9%)	16 (29.1%)	4 (14.3%)
Safety or security reasons	71 (40.8%)	18 (32.7%)	10 (35.7%)
Car ownership	70 (40.2%)	13 (23.6%)	13 (46.4%)
Weather conditions (hot, cold, rainy, sandstorm, etc.)	104 (59.8%)	27 (49.1%)	17 (60.7%)
Long walking time to the campus	78 (44.8%)	18 (32.7%)	7 (25%)
Need to offer rides to other family members or colleagues	38 (21.8%)	4 (7.3%)	5 (17.9%)
Need to carry goods	26 (14.9%)	10 (18.2%)	5 (17.9%)
Lack of primary services and facilities or the physical disability arrangements	14 (8%)	4 (7.3%)	2 (7.1%)

### Willingness to transform to ecomobility modes

At the end of the survey, participants were asked to identify their willingness to transform traveling modes to the campus in the case of solving the barriers in Table 11 they responded as summarized in Table 12. In the Table, there were two questions; the first was about their transformation willingness, and the answers were set to be yes, no, and maybe. The third option was squeezed into the survey to discover the area between acceptance and rejection. Some opinions carry a partial readiness but not enough to decide the acceptance, and these responses were present at the option “maybe.” The study assumes that the last answer could be added to the first one, “yes,” in the case of enhancing the environment’s quality. Accordingly, respondents stated their willingness to transform by 77, 65.4, and 75% of students, faculty members, and staff, respectively. The results of this part give a positive indication for the community response to transform to the ecomobility modes. For the sake of collecting more details regarding the preferable transformation modes, the second question gave options for other public motorized means, “passenging” (bus and car-sharing), and the non-motorized options, especially for the middle and short distances (cycling, wheeling, and walking). The responses showed a potential preference for the bus option as 51.1, 49.1, and 53.6% of students, faculty members, and staff, respectively, stated. The second preference was car sharing, as stated by 29.3, 32.7, and 25% (the previous order). For the non-motorized options, 28.1, 18.4, and 21.8% of students preferred cycling, wheeling, and walking, respectively. Where 18.2, 5.5, and 27.3% of faculty members were their preferences for the same means. 14.3, 10.7, and 17.9% of the staff expressed their preferences for the same options. Analyzing the last results, the students preferred cycling and wheeling means over walking, while faculty members and staff preferences went to the opposite; walking is the first preference over cycling and wheeling.

**Table 12:** Willingness to transform traveling modes to the campus.

Mode\Group		Students	Faculty	Staff
N		174	55	28
Willingness to transform	Yes	59 (33.9%)	23 (41.8%)	10 (35.7%)
	No	40 (23%)	19 (34.5%)	7 (25%)
	Maybe	75 (43.1%)	13 (23.6%)	11 (39.3%)
Preferable transformation mode/s	Bus	89 (51.1%)	27 (49.1%)	15 (53.6%)
	Car sharing	51 (29.3%)	18 (32.7%)	7 (25%)
	Cycling	49 (28.1%)	10 (18.2%)	4 (14.3%)
	Wheeling	32 (18.4%)	3 (5.5%)	3 (10.7%)
	Walking	38 (21.8%)	15 (27.3%)	5 (17.9%)

### Assessment of street’s environment quality

As mentioned in the methods section, the second part of this study was performed by an expert panel as a field assessment to evaluate the environment’s pedestrian quality around the university campus. Table 13 summarizes the results of this field assessment depending on the expert panel assessment of 40 location points distributed among different places within the study area among the P and M street types.

**Table 13:** Expert panel summary results of the streets’ field assessment.

Main criteria	Sub-criteria	Type P streets		Type M streets	
		RII	IL	RII	IL
1. Continuity	a. Pedestrian cont	0.650	H-M	0.350	M-L
	b. Access cont	0.600	H-M	0.300	M-L
	c. Function cont	0.550	M	0.400	M
2. Diversity	a. Vitality	0.650	H-M	0.450	M
	b. Multilayeredness	0.650	H-M	0.350	M-L
	c. Function Variety	0.650	H-M	0.350	M-L
3. Esthetic	a. Visual Qualities	0.650	H-M	0.350	M-L
	b. Attractiveness	0.600	H-M	0.300	M-L
	c. Imageability	0.600	H-M	0.300	M-L
4. Comfort	a. Safety	0.600	H-M	0.350	M-L
	b. Access comf	0.650	H-M	0.300	M-L
5. Scale	a. Human	0.600	H-M	0.300	M-L
	b. Environmental Compliance	0.600	H-M	0.300	M-L

According to Table 3, type P streets achieved greater results than type M. The field assessment confirmed that the environment quality to accommodate ecomobility modes differs from one location point to another, with averages indicating P's dominance over M street types.

## Discussion

This section revises the collected results in the last part, giving more profound readings of the respondents’ survey choices and the research progress in this field. Likewise, the field streets’ assessments are discussed intensely to understand the status and possible future enhancements.

### Principal transportation modes

Considering different barriers like culture, weather, and distance, the walking mode was chosen as a transportation mode by only 2% of the respondents. Likewise, the assessment of the pedestrian environment’s quality around the campus indicated a lack of cycling facilities, which justifies not choosing the cycling mode by the respondents. Compared with a previous study for the same PSU community in 2021^15^, there was no use of electric or hybrid cars, but there was a dependency on these cars in the current study (0.8%, and 5.4% for males and females, respectively). This dependency is a positive indication for the transformation towards clean and green energy resources that seems promising soon. Also, it is worth mentioning that the percentage of owning private cars for females is ascending compared with males: the previous 2021 study indicated that these percentages were 77.6% and 60.9% for males and females, respectively, which demonstrated the domination of males in this indicator. However, in the current study, the female percentage has exceeded the male one by 4.3%. This rise looks reasonable concerning the Saudi permit for females to get driving licenses after being banned before, which is also expected to rise in the following years. On the other hand, the public transportation comprehensive project, including Metro and bus with different categories, is not started yet and is expected to initiate during the current year. However, several bus lines partially operate in some selected areas for testing purposes, which justifies the low current dependency on the public bus. Still, the current status of traveling modes is below the global levels due to the mentioned reasons, e.g., lack of bicycle lanes, the slow transformation to electric cars and other relevant transportation facilities, the adopted walking environment in hot weather areas, and the overall culture of the public to deal with the environmental challenges due to their cumulative habits.

### Factors controlled choices

According to the results in “Results /Factors controlled choices”, only 3.6% of the staff who checked the choice of “not owning a car have a driving license.” This low dependency maybe refers to the 67.9% of citizens who form this group and mostly own private cars. For the private bus, the faculty members’ group was the highest number of users. It is justified as the University offers its bus as an option with a transportation allowance. This option is almost used by the non-Saudis (especially non-Arabic speakers), whose culture prefers the economic and practical options. Likewise, the staff members who use the bus are from the same nationalities (citizens). Diverse groups are users of other transportation modes rather than driving. Those groups included reasons like the temporary unavailability of their cars due to maintenance or not owning cars, or driving licenses. Some others seek Convenience and well-organized university bus rides either for the regular paths between the home and the campus or for shopping.

On the other hand, walking mode choices are summarized in Table 10. Students, and faculty, agreed that the short travel distance to the campus was the reason behind choosing this option. However, the walking mode choice was limited to two groups and low intensity (1.1 and 3.6%) of students and faculty members, respectively. The last finding agreed with the one in a previous study performed on a similar city in a high-income developing country^50^. The culture of wealthy countries’ public offers the dominance of psychological comfort using private and big cars at the expense of other means of transportation that consider the people's health and clean environment. This culture needs more time to shift, and the country's role is to encourage people to transform to ecomobility modes through regular campaigns and reinforce the targeted transportation infrastructure.

### Barriers of the ecomobility modes

Low dependencies on walking and cycling modes were justified by the unavailability of bike lanes or continuous sidewalks. The last results agreed with other previous studies’ findings, which considered the lack of pedestrians’ safety and comfort facilities a substantial barrier to walking and cycling modes^51,52^. Weather conditions, trip time, and long walking time to the campus were significant reasons to avoid this mode. In general, stating the barriers to using the clean and green means of transportation gives a positive indication of the possible transformation to ecomobility modes. This transformation could occur in parallel with the continuous governmental efforts to accomplish the country’s 2030 vision of much more sustainable actions for a better environment and human health. It is worth mentioning that the service quality influences the intention to use less driving and more use of other sustainable means of transportation^53^.

### Willingness to transform to ecomobility modes

Car sharing, one of the ecomobility modes (passenging), was the second-highest chosen option by all groups. Comparing with the same groups’ responses in Table 8 (21.3, 16.4, and 3.6%) for the current dependency on car sharing, it’s expected to have a rise of 8, 16.3, and 21.4%, respectively, which represents the expected willingness to replace the current driving option to one of the passenging means of transportation. For other modes, as mentioned in 3.4, students preferred cycling and wheeling means over walking, while faculty members and staff preferred walking over cycling and wheeling. These results are justified by the age factor as older people prefer walking as the superior physical exercise, while cycling and wheeling suit younger ones more. Further than the survey results, it is believed that after removing or minimizing the barriers to ecomobility transformation, the shown percentages will even change positively, considering the culture change that encourages more people to have a different mindset serving the preferable environmental modes.

### Assessment of environment’s quality of streets

**Figure 7:**
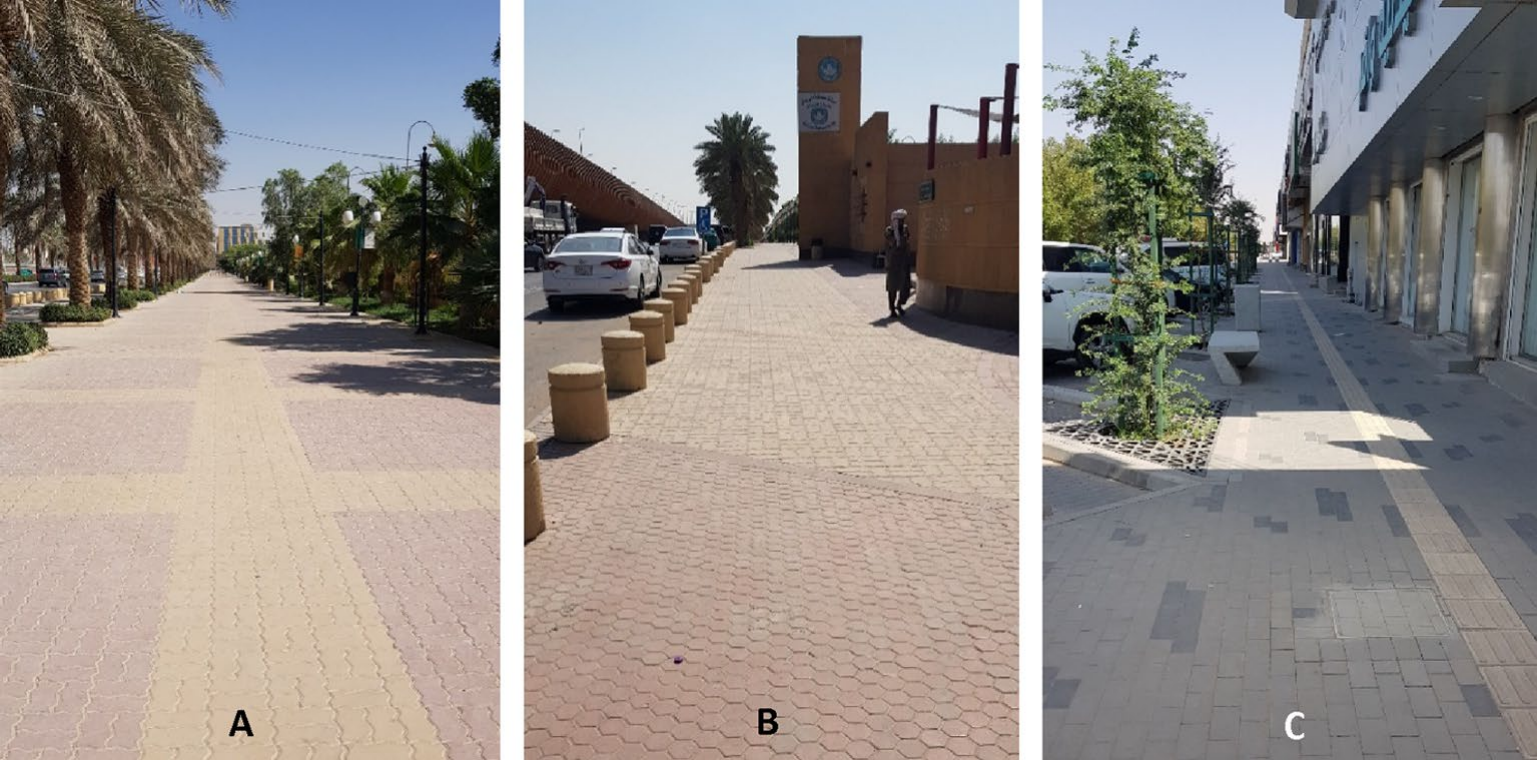
Examples for the street status “good for mobility” of type P, where (**A**, **B**), and (**C**) are for location points 4, 5, and 11, respectively (See Fig. 5).

**Figure 8:**
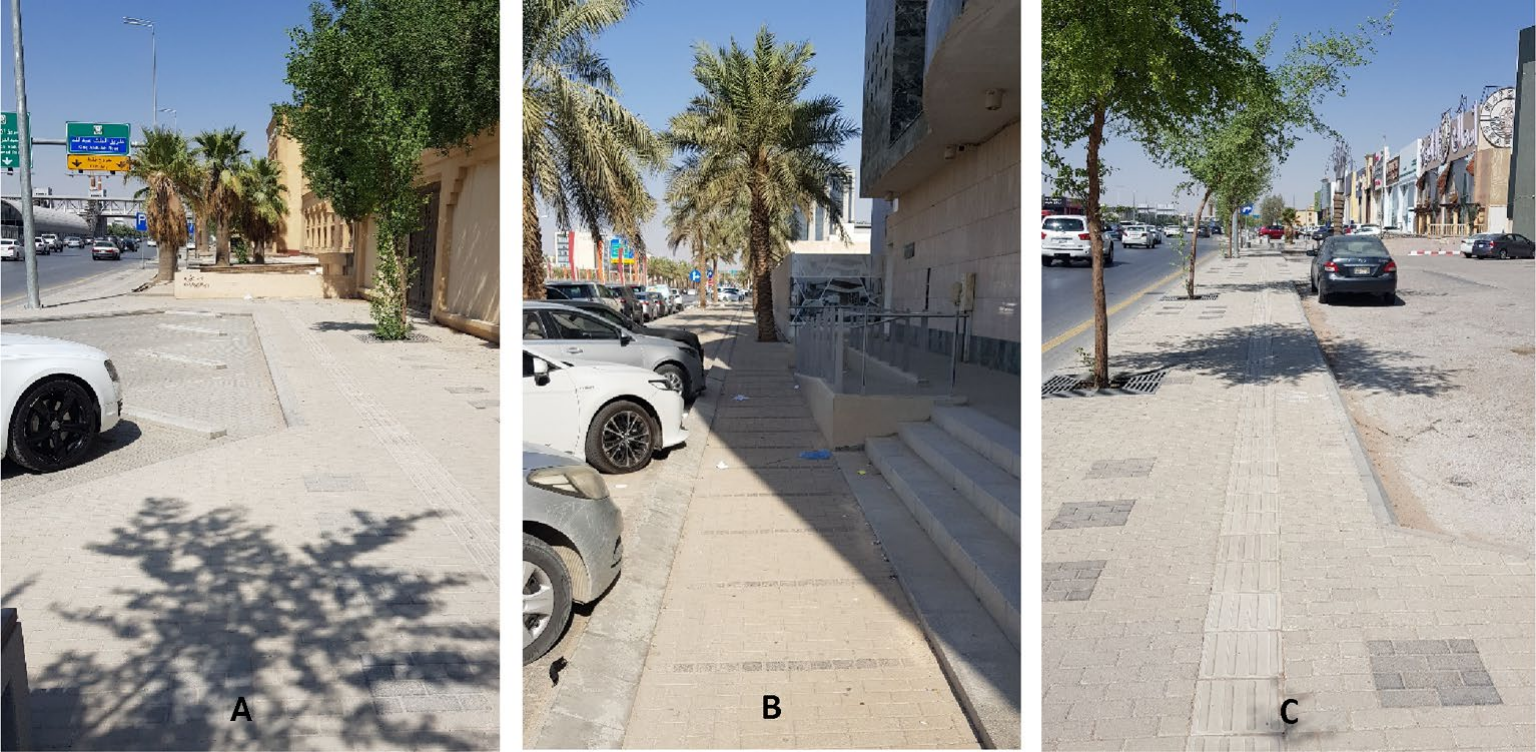
Examples for the street status “Passable for mobility but requires minor attention” of type P, where (**A**, **B**, **C**) are for location points 1, 6, and 20, respectively (See Fig. 5).

**Figure 9:**
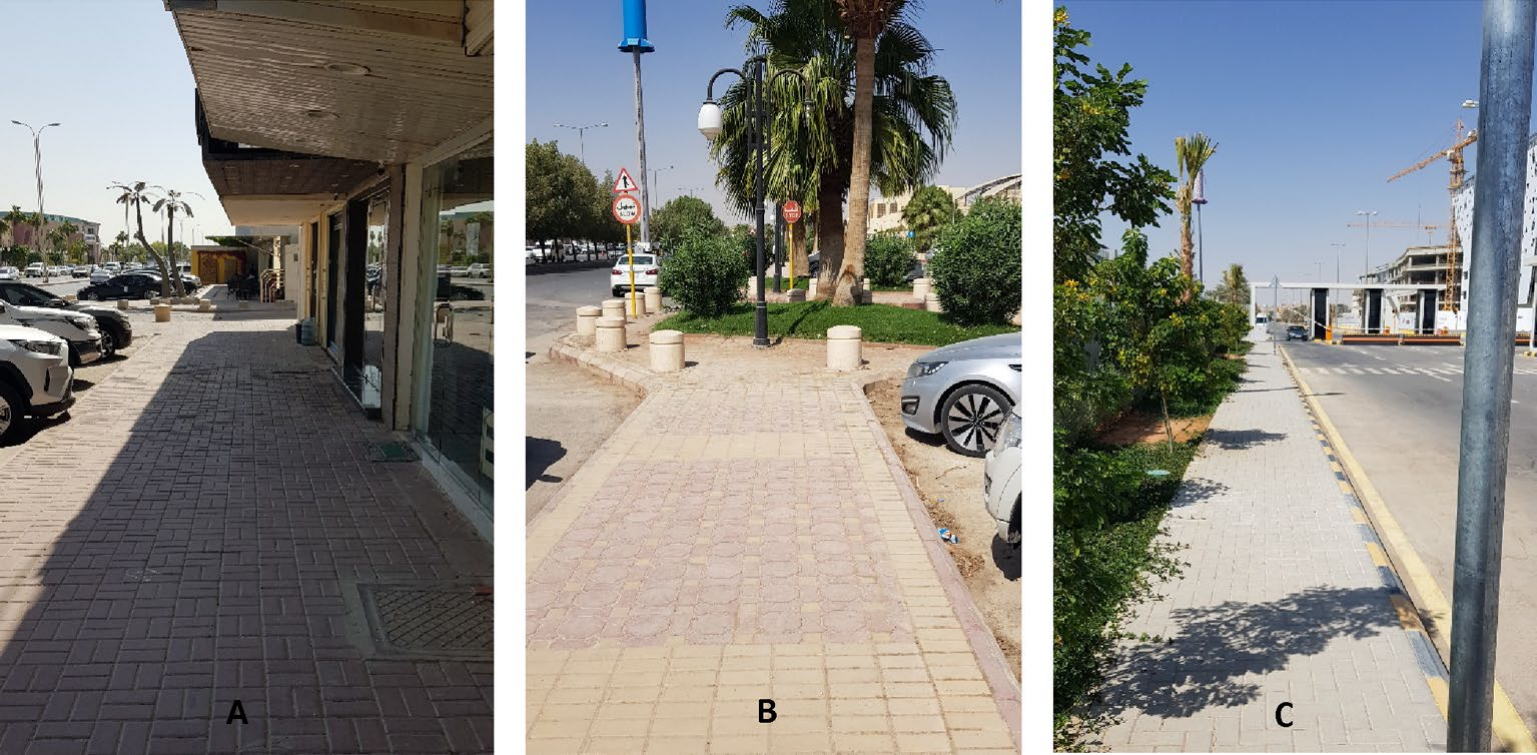
Examples for the street status “Passable for mobility but requires minor attention” of type M, where (**A**, **B**, **C**) are for location points 1, 3, and 19, respectively (See Fig. 5).

**Figure 10:**
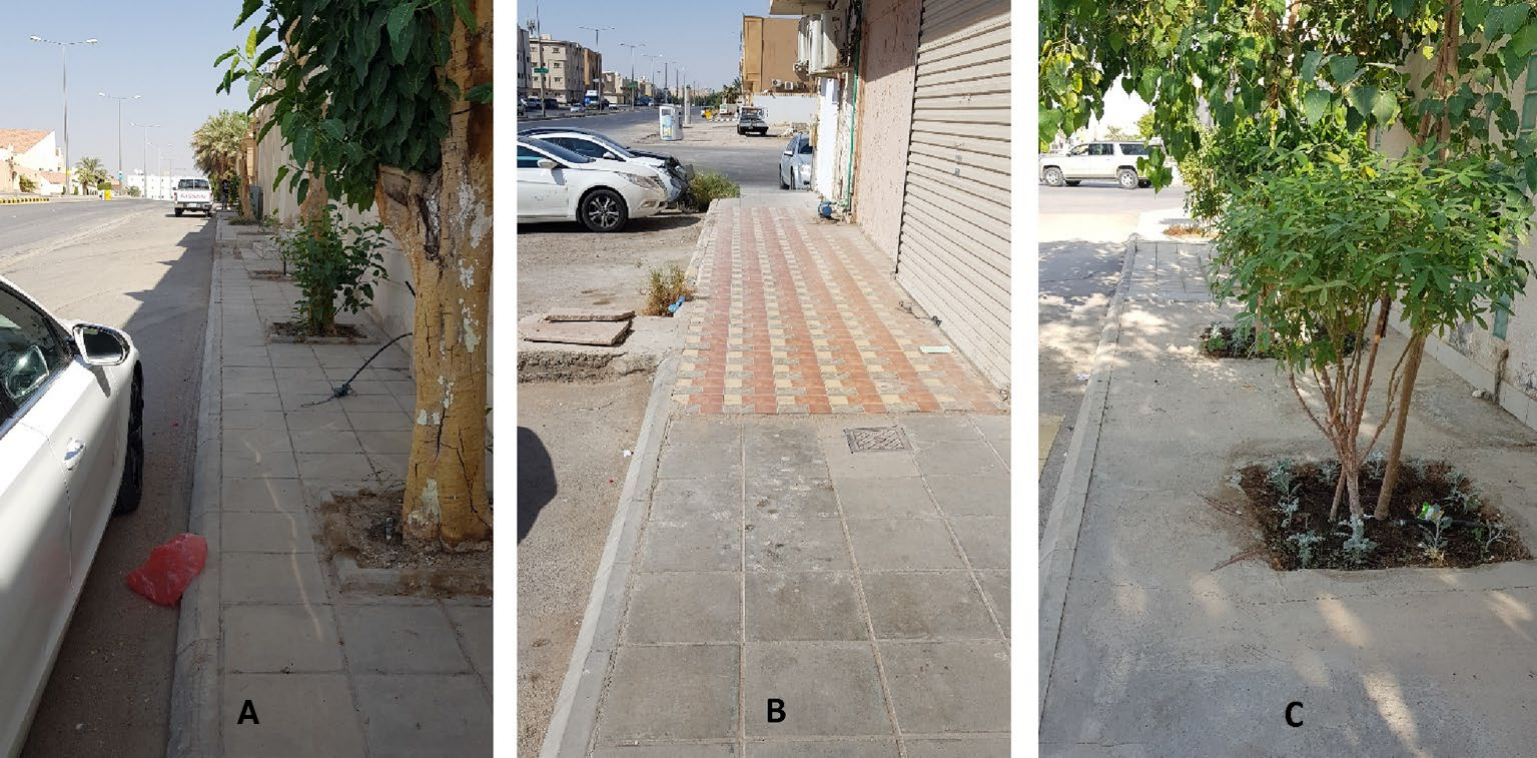
Examples for the street status “insufficient for mobility and requires some attention” of type M, where (**A**, **B**, **C**) are for location points 10, 15, and 18, respectively (See Fig. 5).

The average IL of type P is High-Medium (H-M, except for function continuity: M), whereas the same IL of type M is Medium–Low (M-L, except for function continuity and vitality: M). The “function continuity” is assessed as a medium in both types regardless of the averages of other items of the sub-criteria, which means that it’s the weakest point in type P, and one of the strongest points in type M. In other words, the “function continuity” needs consideration in both types, especially for type P (Passable for mobility but requires minor attention). Figures 7, 8, 9, 10, give examples of the assessed location points among both types, where Figs. 7, 8 are for “good for mobility” and “Passable for mobility but requires minor attention” of type P streets, respectively. On the other hand, Figs. 9, 10 are “passable for mobility but requires minor attention” and “insufficient for mobility and requires some attention” of type M, respectively. It is highly expected that governmental enhancements to significant sectors, including transportation facilities (achieving the related SDGs up to the 2030 vision), will change many pedestrians’ traveling modes, which serve the total transformation to ecomobility modes.

## Conclusions

This study assessed and investigated pedestrians’ traveling modes in Riyadh city. The ecomobility was evaluated through a case study at Prince Sultan University (PSU) and its surrounded environment. The achieved assessment was divided into two approaches using a web-based survey and field assessment for streets to evaluate the environment’s quality that assists pedestrians’ transformation to ecomobility modes. The environment quality around the campus was evaluated per well-supported criteria (five main and 13 sub-criteria) held by an expert panel among 40 location points distributed on street types P and M. This study uncovered the pedestrian’s possible transformation modes towards ecomobility by defining the targeted factors of the livable streets around the PSU campus in Riyadh city. Also, it emphasized the need to promote the pedestrian’s ecomobility concept throughout everyday life, according to available options. The study noticed that the current mobility mode extensively relies on private fossil-fueled cars. A considerable percentage of the survey sample (91%: 88% fossil-fuel cars, and 3% electric or hybrid cars) is driving to the University. Nevertheless, the assessment results showed that the transformation to ecomobility is expected by the entire operation of the public bus project in Riyadh and the continuous governmental enhancements to the quality of the street environment. Around 72.5% of the survey respondents expressed willingness to transform to ecomobility modes (including “Yes and Maybe” responses). The study also revealed that about 51.3% of the survey sample preferred the public bus option in the case of achieving the expected transformation. For the non-motorized options, students preferred cycling and wheeling modes, while faculty members and staff preferred walking. The first two modes fit the younger ages more than walking, including older generations. The study sample referred to the lack of compliance to the ecomobility means of transportation to the barriers. The research assumes that handling the barriers through future governmental plans will solve this point, as will be introduced in the next section. The pedestrians’ willingness to transform to the ecomobility traveling modes will grant touchable enhancements to the health and the environment. It will provide better health, clean air, mobility and accessibility, noise avoidance, reduced greenhouse (and CO_2_) gas emissions, individual cost savings, and energy efficiency.

### Recommendations

Complying with the 2030 vision, the Saudi government is keen on achievement of the SDGs 13, 3, and 11, which are related to the clean and green environment, better health, clean air, mobility and accessibility, noise avoidance, greenhouse gas emission reduction, individual cost savings, and energy efficiency. In accordance, the Saudi government should prioritize development objectives to assist citizens and residents in contributing to ecomobility mode, i.e., promotion integration of cycling, walking, passenging, and wheeling. This contribution will help achieve a more sustainable, livable, clean, and green environment. Through this approach, the results will go for less dependency on driving (especially the fossil-fuel cars) and more use of passenging, and non-motorized transportation means, e.g., cycling, wheeling, and waking. Therefore, the community will benefit from carpooling, a clean and green environment, and better health. The research, through its two assessment parts, can recommend the following actions:

- Add more shading facilities and plants to improve the street environment. This addition may encourage pedestrians to use non-motorized transportation means, especially wheeling and walking, enhancing community health.
- Include cycling lanes in the suitable street types at the expense of the relatively wide sidewalks in type P streets.
- Invest in the succession of using the university buses for faculty members and staff by offering this service to students. This step could reduce the current spacious parking areas inside the campus, solve traffic and congestion problems, and be a good step towards ecomobility.
- Employ related Geo-Thermal technologies systems to overcome the adverse weather troubles, which is a significant barrier to using non-motorized transportation modes.

### Limitations and future studies

The study assessments were implemented between October and November 2021, close to the end of strict governmental precautions due to Covid-19 Pandemic, and during the experimental operation phase of some new public bus lines. For future research, the paper unveils an approach that could be employed to assess the pedestrian’s traveling modes after the full operation of the comprehensive transportation project in Riyadh for bus and Metro. On the other hand, street types R and A that were not covered through this study could be assessed in future research.

## Data Availability

Some or all data, models, or code that support the findings of this study are available from the corresponding author upon reasonable request. (Survey responses and statistics, Expert panel street assessments, RII calculations).
